# Site-Specific N-Glycan Characterization of Grass Carp Serum IgM

**DOI:** 10.3389/fimmu.2018.02645

**Published:** 2018-11-14

**Authors:** Yi-Ling Su, Bing Wang, Meng-Die Hu, Zheng-Wei Cui, Jian Wan, Hao Bai, Qian Yang, Yan-Fang Cui, Cui-Hong Wan, Li Xiong, Yong-An Zhang, Hui Geng

**Affiliations:** ^1^Hubei Key Laboratory of Genetic Regulation and Integrative Biology, School of Life Sciences, Central China Normal University, Wuhan, China; ^2^State Key Laboratory of Freshwater Ecology and Biotechnology, Institute of Hydrobiology, Chinese Academy of Sciences, Wuhan, China; ^3^College of Modern Agriculture Sciences, University of Chinese Academy of Sciences, Beijing, China; ^4^Key Laboratory of Pesticide and Chemical Biology, Ministry of Education, Central China Normal University, Wuhan, China; ^5^State Key Laboratory of Agricultural Microbiology, Huazhong Agricultural University, Wuhan, China; ^6^Laboratory for Marine Biology and Biotechnology, Qingdao National Laboratory for Marine Science and Technology, Qingdao, China

**Keywords:** teleost, grass carp, immunoglobulin M, N-glycan, liquid chromatography-electrospray ionization tandem mass spectrometry (LC-ESI-MS/MS), glycosylation, matrix assisted laser desorption/ionization-time-of-flight-MS (MALDI-TOF-MS)

## Abstract

Immunoglobulin M (IgM) is the major antibody in teleost fish and plays an important role in humoral adaptive immunity. The N-linked carbohydrates presenting on IgM have been well documented in higher vertebrates, but little is known regarding site-specific N-glycan characteristics in teleost IgM. In order to characterize these site-specific N-glycans, we conducted the first study of the N-glycans of each glycosylation site of the grass carp serum IgM. Among the four glycosylation sites, the Asn-262, Asn-303, and Asn-426 residues were efficiently glycosylated, while Asn-565 at the C-terminal tailpiece was incompletely occupied. A striking decrease in the level of occupancy at the Asn-565 glycosite was observed in dimeric IgM compared to that in monomeric IgM, and no glycan occupancy of Asn-565 was observed in tetrameric IgM. Glycopeptide analysis with liquid chromatography-electrospray ionization tandem mass spectrometry revealed mainly complex-type glycans with substantial heterogeneity, with neutral; monosialyl-, disialyl- and trisialylated; and fucosyl-and non-fucosyl-oligosaccharides conjugated to grass carp serum IgM. Glycan variation at a single site was greatest at the Asn-262 glycosite. Unlike IgMs in other species, only traces of complex-type and no high-mannose glycans were found at the Asn-565 glycosite. Matrix-assisted laser desorption ionization analysis of released glycans confirmed the overwhelming majority of carbohydrates were of the complex-type. These results indicate that grass carp serum IgM exhibits unique N-glycan features and highly processed oligosaccharides attached to individual glycosites.

## Introduction

Glycosylation represents a major post-translational modification of proteins involved in various biological processes, including transcription, differentiation, apoptosis, cell adhesion, receptor-ligand binding, as well as oncogenic transformation and immune response (1–4). Almost all proteins in the immune system are glycoproteins, the attached glycans are thought to be crucial to their structure and the immune effector mechanism (5, 6).

The two main types of glycan linkages to proteins are the N-linked and O-linked types. The N-linked oligosaccharide is covalently bonded with nitrogen of asparagine when it occurs in the sequence Asn-X-Ser/Thr or, more rarely, as part of an Asn-X-Cys motif (where X≠Pro) (7). N-linked oligosaccharides have been classified into three most common ones, being the high-mannose, hybrid, and complex types. All have a basic core structure of conserved pentasaccharide (GlcNAc_2_Man_3_) backbone but vary with respect to the structures attached to this core (8).

The N-linked oligosaccharides presenting on immunoglobulins have received particular attention because changes in the attached glycans can impact immunoglobulin solubility, structural stability, and biological function (9). In human IgG, differential of single monosaccharide at Asn-297 glycosite at the Fc fragment can drastically affect IgG binding to FcγR (10, 11) and affecting complement action (12). In human IgM, there are five putative glycosylation sites (Asn-171, Asn-332, Asn-395, Asn-402, and Asn-563) on the heavy chain. The glycans linked to each glycosites of IgM have been demonstrated to be involved in various biological functions, including B-cell maturation (Asn-171) (13), complement activation (Asn-402) (14, 15), and J-chain incorporation (Asn563) (16, 17).

In teleosts, IgM is the major antibody in serum, and it plays a key role in humoral adaptive immunity. Similar to mammalian IgM, teleost IgM consists of two identical heavy and two identical light chains (2H+2L). The heavy chain possesses four constant domains, CH1–CH4, containing the sites for the binding of effector cells (18), cytotoxic cells (19), or molecules such as complement system components (20). Bioinformatics analysis of teleost IgM has indicated the presence of 4–5 N-linked glycosylation sites in CH2, CH3, and CH4, while there is no glycosylation at CH1 (21, 22). The reported carbohydrate content was estimated to be approximately 12.5% for Atlantic salmon (*Salmo salar*) and 10% for Atlantic cod (*Gadusmorhua*) IgM (23, 24). Atlantic cod IgM contains oligosaccharides with N-acetylneuraminate and a core-fucose unit (24). A study of the IgM of the nurse shark (*Ginglymostomacirratum*), a cartilaginous fish, revealed that the most abundant glycan was high-mannose type. Moreover, the structures of both complex glycans and high-mannose on nurse shark IgM were very similar to the vertebrate species (25).

Compared to mammalian IgM, teleost IgM displays a significant degree of structural diversity, with different polymerization states, including monomers, dimers, trimers, and tetramers, being revealed (26–29). Structural diversity appears universal among teleost IgMs, the cause of the structural diversity has remained elusive for decades. The C-terminal tailpiece of secretory IgM is highly conserved, cysteine residue and a N-glycan Asn-X-Ser/Thr sequence have been found in all species sequenced thus far, including human (30), mouse (31), bovine (32), rabbit (33), chicken (34), hamster (35), frog (36), and fish (22). The C-terminal tailpiece contains crucial information for mammalian IgM assembly, as IgM polymers are influenced by the presence of glycans linked to Asn-563 in human IgM (37, 38). Furthermore, glycans linked to conserved glycosite at the C-terminal tailpiece has been demonstrated to influence the binding of joint chains and also mammalian IgM polymerization (16). A recent report revealed that replacement of Asn-563 with alanine (Ala) in human IgM prevents the attachment of N-glycans on IgM, resulting in the secretion of higher molecular weight hexameric, rather than pentameric, IgM complexes. This suggests that Asn-563 glycans at the C-terminal tailpiece play important roles in the process of IgM polymerization (39). Taken together, these findings indicate that glycosylation at conserved C-terminal tailpieces maybe an interesting field of study for analyzing the incorporation and formation of polymeric IgM differences in teleosts.

Thus far, however, no data have been reported on the site-specific N-glycan of teleost IgM. In order to characterize these site-specific N-glycans, we conducted the first comprehensive study of the carbohydrates conjugated at each N-glycosylation site, as well as the occupancy at the Asn-565 residue in the conserved C-terminal tailpiece, among grass carp IgM isoforms. These data expand the limited information currently available concerning teleost IgM glycosylation and provide a knowledge base for understanding the biological significance of glycosylation on teleost IgM.

## Materials and methods

### Analytical approach

**Figure 1:**
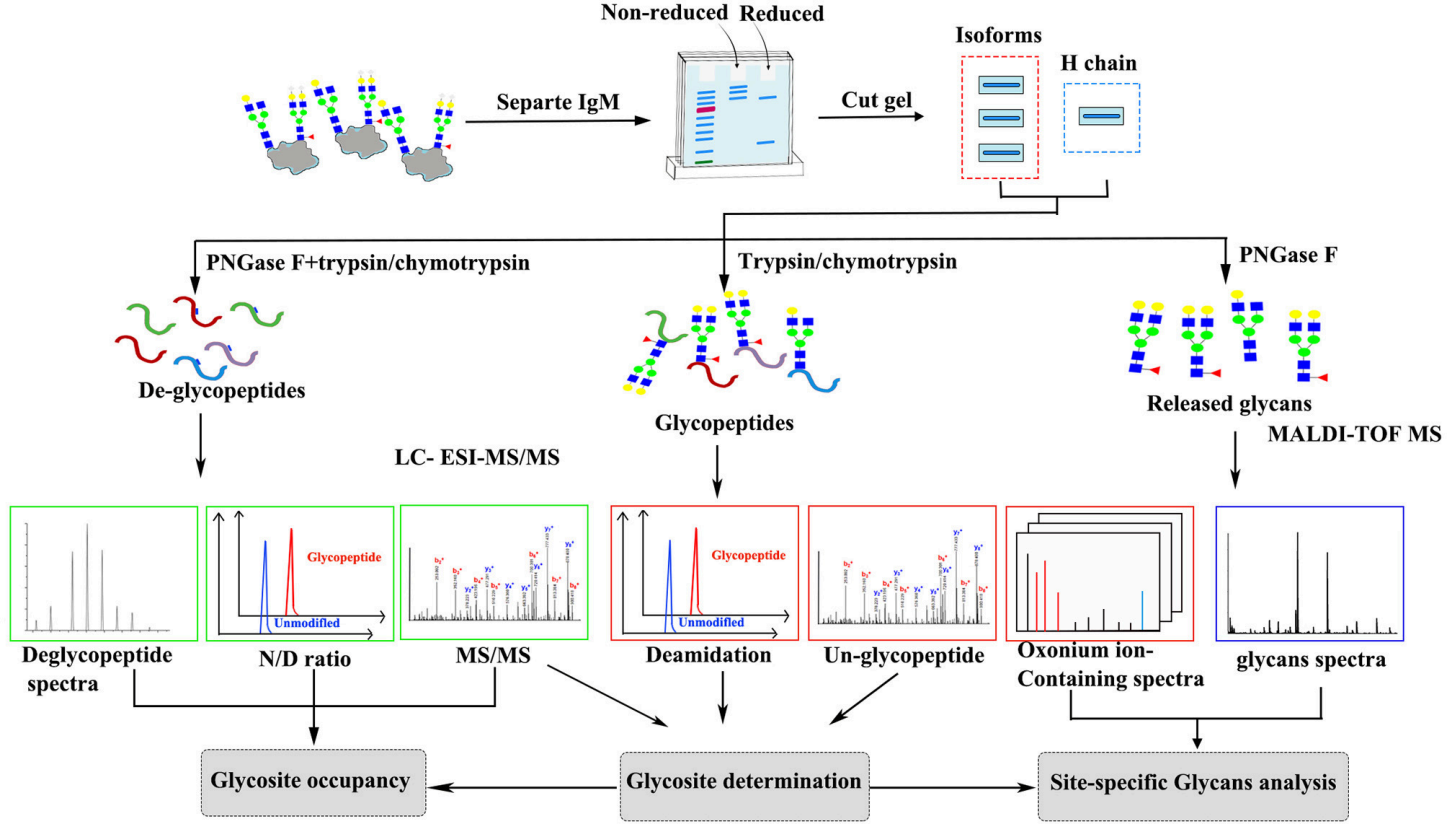
Scheme of the experimental approach to characterize grass carp serum IgM glycoform.

The general approach to characterizing IgM glycosylation structures is shown in Figure 1. First, liquid chromatography-electrospray ionization tandem mass spectrometry (LC-ESI-MS/MS) was used to map tryptic/chymotryptic deglyco-peptides and to identify the N-linked glycosylation sites. N-glycan occupancy at Asn-565 residues between monomeric, dimeric and tetrameric IgM were determined by comparing de-glycosylated and non-glycosylated N-565 peptides peak areas. LC-ESI–MS/MS analysis with in-source higher energy collision dissociation (HCD) was used to locate glycopeptides and for site specific glycoform characterization. Glycoform characterization was further confirmed by MALDI-TOF MS analysis of release glycans.

### The fish and serum collection

Grass carp (Ctenopharyngodonidella idella) 350–500 g, were bred and kept in 1,000-L tanks with recirculating and Uv-sterilized water. Fish were anesthetized with 10 ppm Benzocaine (Beijing, China National Pharmaceutical Group Corporation) for 3–5 min, blood was collected from the caudal vein from 10 naïve grass carp, allowed to clot for 1 h at room temperature and overnight at 4°C, serum was collected after centrifuging at 4,000 rpm for 10 min, pooled and stored at −80°C. All animal experiments were approved by the Institutional Animal Care and Use Committee of Central China Normal University.

### Preparation of IgM

IgM was purified from pools of 10 individuals using a combination of polyethylene glycol (PEG) precipitation and ion-exchange chromatography as described previously (40). Briefly, Grass carp plasma was precipitated by added 16% (w/v) PEG (final concentration), stirring at 4°C for 30 min. The precipitated proteins were collected by centrifugation at 10,000 × g for 15 min. The collected protein were dialyzed against 10 mM sodium phosphate buffer (pH 7.5) containing 5 mM EDTA at 4°C overnight, the dialyzed buffer was changed for 3 times. The protein samples were passed through Source Q anion column (Amersham Biosciences) with 10 mM sodium phosphate buffer (PBS 0.01 M, pH 7.5) containing 5 mM EDTA. Bound proteins were eluted with a 0–1M linear NaCl gradient at a flow rate of 1 ml/min. Fraction containing grass carp IgM was collected and dialyzed against 10 mM sodium phosphate (pH 5.9), and further polished by pass through Source S cation exchange resin (Amersham Biosciences). Purity and size of IgM were checked by SDS-PAGE analysis and Coomassie Brilliant Blue G250 staining.

### Gel electrophoresis

To isolate the heavy and light chains of grass carp IgM, 4 ug of purified IgM was mixed with denatured loading buffer and loaded onto 4–10% gradient SDS-polyacrylamide gel, then subjected to electrophoresis at 25 mA till the dye reached the bottom of the gel. The gel was stained with Coomassie Brilliant blue (Sigma) and the heavy and light chains was determined according to molecular weight markers. Previously electrophoretic analysis revealed carp fish serum containing different populations as monomeric, dimeric, and tetrameric forms of IgM (41, 42). To verify of the oligomerization state of the grass carp serum IgM, 8 ug of purified IgM was mixed with loading buffer without β-mercaptoethanol and characterized by 6% SDS-polyacrylamide gel electrophoresis at 30 mA for about 2.5 h. The gel was stained with Coomassie Brilliant blue and the molecular band of monomer, dimer, and tetramer was estimated.

### Glycosylation site determination

To determination the glycosylation site on grass carp IgM, coomassie-stained SDS-PAGE gel pieces containing IgM heavy chain were destained with a solution of 50 mM ammonium bicarbonate in 50% acetonitrile (1:1, vol/vol) with shaking, and followed by 100% acetonitrile. The sample pieces were reduced by incubating with 64 mM DTT at 56°C for 60 min, followed by alkylation with 130 mM iodoacetamide at R.T. for 45 min in the dark. The samples were washed with 50 mM ammonium bicarbonate and dehydrated in acetonitrile, dried in a SpeedVac centrifuge, rehydrated in 50 mM ammonium bicarbonate. N-glycans were released by incubating the sample with 100 Units/ml of PNGase F for 12 h at 37°C, the N-linked glycans were collected for further MALDI-TOF analysis. Gel piece was dehydrated in acetonitrile, rehydrated in 50 mM ammonium bicarbonate and treated with trypsin (Promega) at an enzyme/substrate ratio of 1:20 (w/w) or with chymotrypsin (Promega) at an enzyme/substrate ratio of 1:13 (w/w) overnight at 37°C. Peptides were extracted from the gel pieces with 50% acetonitrile in 5% formic acid. The resulting peptides were then extracted and desalted with a C18 tip (Millipore) for analysis by LC-ESI-MS/MS. Glycosylation site were determined by mass of deglycopeptide-peptide (Asn to Asp; Δ*m* = +0.984 Da). Sequence and potential N-glycosylation sites for grass carp serum IgM were obtained from GenBank (accession No ABD76396.1).

### N-glycopeptide enrichment by HILIC

The digested peptides were enriched with Unisol-Amide HILIC media as previously described with slight modifications (29, 43). Briefly, the gel band corresponding to the IgM heavy chain (approximately 4 μg) was excised, destained and digested with trypsin (Promega) or chymotrypsin (Promega) overnight. Unisol-Amide HILIC resin (Agela, China, 10 μm particle size, 200 Å pore size) were packed on top of a 200 μL C18 tip (Millipore) to an approximate column height of 5 mm. The column was washed with 1% formic acid followed by 1% formic acid in 80% acetonitrile. The trypsin or chymotrypsin digested peptide mixtures were dissolved with 20 μL 80% acetonitrile and loaded onto the column, the flow through fraction was reloaded onto the column twice to maximize the glycopeptide binding, the column was then washed thrice with 1% formic acid in 80% acetonitrile. The bound glycopeptides were eluted twice with 1% formic acid, and subsequent elution with 20 μL 1% formic acid in 80% acetonitrile was performed to elute any glycopeptides binding to the C18 tip during the elution. The enriched glycopeptides were dried and kept at −20°C till for analysis.

### LC-ESI-MS/MS analysis

Trypsin or chymotrypsin digested grass carp IgM de-glycopeptides and glycopeptides were analyzed using nanoflow HPLC instrument (EASY-nLC 1200 system, Thermo Fisher Scientific, USA) coupled on-line to a Q Exactive Plus mass spectrometer (Thermo Fisher Scientific, USA) in positive polarity mode. Chromatography column was Acclaim PepMap, C18, 50 um × 150 mm, particle size 2 um, 100Å (Thermo Fisher Scientific, USA) with buffer A (95% water and 4.9% acetonitrile with 0.1% FA) and buffer B (90% acetonitrile and 9.9% water with 0.1% FA) at a flow rate of 300 nL/min. The gradient profile was set as follows: 3–5% B for 6 min, 5–25% B for 54 min, 25–40% B for 25 min and hold 5 min, 40–80% B for 5 min, and 80–100% B for 5 min. The electrospray voltage was 2.0 kV. Peptides were analyzed with high-energy collisional dissociation (HCD) fragmentation mode by data-dependent MS/MS acquisition. In MS1, the resolution was 70,000, the AGC target was 3e^6^, and the maximum injection time was 20 ms. In MS2, the resolution was 17,500, the AGC target was 5e^4^, and the maximum injection time was 50 ms, NCE stepped set 27. The scan range was set at a resolution of 350–2,000 m/z and the 20 most intense precursors were chosen analysis by data-dependent mode.

### Analysis of De-glycopeptide and intact glycopeptide MS data

N-linked-glycosylation sites were determined in de-glycosylated peptides based on converting Asn to Asp by PNGase F treatment, the corresponding glycosylated peptides were confirmed by HCD tandem MS. The obtained de-glycopeptide MS data of were searched against grass carp IgM sequence (GenBank accession No ABD76396.1) using Proteome Discoverer V2.2 (Thermo Fisher Scientific). Carbamidomethylation of Cys was set as a fixed modification, oxidation of Met and converting of Asn to Asp were set as variable modifications. The mass tolerance was set at 10 ppm for precursor ions, and the MS/MS mass tolerance was set at 0.01Da. Trypsin or chymotrypsin was set with up to two missed cleavages. Annotation of the corresponding glycopeptide spectra was performed in using Byonic software v1.09 (Protein Metrics) (44). Searches were performed with the following modifications: precursor ion tolerance of 10 ppm, carbamidomethylation of Cys, protease treatment with up to two missed cleavages, and with the following variable modifications: oxidation of Met and glycosylation of Asn. The obtained grass carp IgM glycopeptide MS data were identified by Byonic software (44). Additionally, all assigned N-glycopeptide HCD spectra were then queried to ensure the presence of diagnostic oxonium ions at m/z 204.087 (HexNAc^+^), and at least one of the following oxonium ions as 186.076 (HexNAc-H2O^+^), 168.066 (HexNAc-2H2O^+^) and 366.140 (HexNAc-Hex^+^) within 50 ppm. The existence of peptide or peptide+HexNAc ions in MS/MS spectra was checked manually to determine the glycosite-containing peptide and glycan compositions of the glycopeptides. Spectra were also checked manually about b and y ion data from peptide portion of the corresponding glycopeptides.

### MALDI-TOF MS analysis glycans

N-glycans released from grass carp serum IgM by PNGaseF treatment were analyzed by an Axima MALDI Resonance mass spectrometer (Shimadzu). N-glycans were dissolve in 10 μl 0.1% TFA followed by spotting 1 μl glycan sample with 1 μl 2,5-dihydroxybenzoic acid matrix (100 mg/ml DHB in 50% acetonitrile, 0.1 mM NaCl) onto a MALDI plate. Positive ion MALDI spectra were obtained with a Bruker ultraflextreme™ Waters-MicromassToF Spec 2E time-of-flight (TOF) mass spectrometer (Waters MS Technology Ltd, Manchester, UK) operated under flexControl 3.3 (Build 108; Bruker Daltonics). Spectra were obtained in positive linear ion mode in the range 1,000–3,200 m/z, using an accelerating voltage of 2.2 kV and a laser energy of 6.5 kV. The positive control in the MALDI-TOF MS analysis was authentic complex-type glycans from mAb rituximab, 2,5-dihydroxybenzoic acid matrix was used as negative control. The average MS spectra (1,000 profiles) were used for glycan assignment by GlycoMod software (http://web.expasy.org/glycomod/) (45). N-glycans identification was set by allowing a maximum mass deviation of 0.04 Da for mainly present [M+Na]^+^ ions. Searches were conducted with the following parameters: Hexose range 3-9, HexNAc range 2-9, deoxyhexose range 0-4, NeuAc range 0-4, NeuGc no, Pentose no, Sulfate/Phosphate no, KDN no, HexA no, UniCarbKB entries were listed separately.

## Results

### Purification of IgM and analysis of N-glycosylation assignments

**Figure 2:**
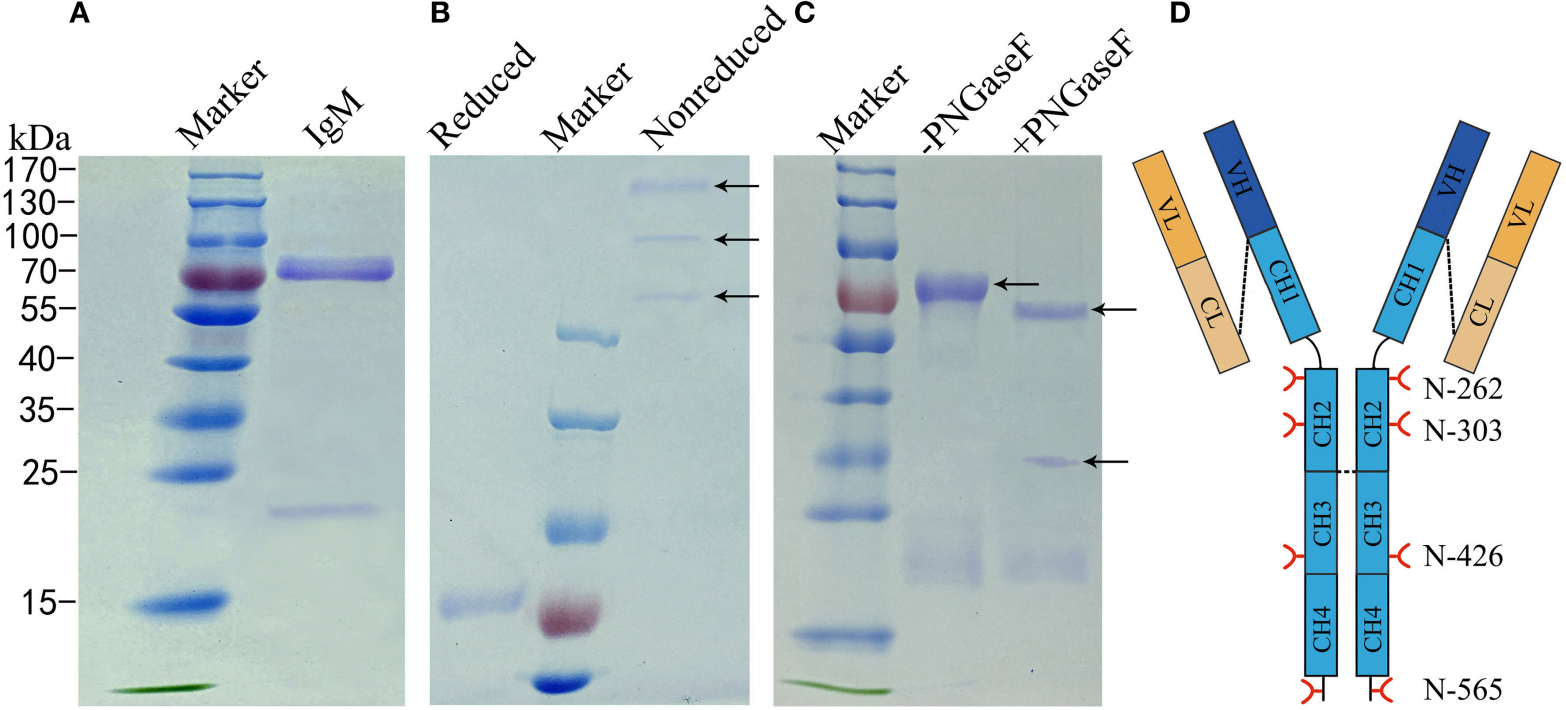
Analysis of N-glycans assignments on grass carp serum IgM. **(A)** A 4–10% uncontinued SDS-PAGE of reduced samples to show 75–80 kDa heavy chain band and 23 kDa light chain band of grass carp IgM. **(B)** A 6% SDS-PAGE stained Coomassie Blue with showing different grass carp IgM isoforms, in the non-reduced samples lane, from up to down, the tetramer, dimer, and monomer were marked by arrow. **(C)** IgM digested with (+) or without (–) PNGase F, arrow at the bottom indicate PNGase F. **(D)** Schematic of four N-glycosyationsites on grass carp IgM, red forks represent four putative N-glycosylation sites on heavy chain, black lines at the end of CH4 domain indicating 16–18 amino acid residue at the C-terminal tailpiece, possible inter-subunit disulfide bridges between heavy chains and covalently link the light chain are shown with black dash lines.

To determine the glycosylation status of grass carp IgM, IgM was purified by polyethylene glycol (PEG) precipitation and ion-exchange chromatography from serum. Purified IgM migrated two major protein bands with molecular weights of 75 kDa and 23 kDa under reducing conditions, representing the heavy and light chains, respectively (Figure 2A). To identify different isoforms of grass carp serum IgM, intact IgM molecules were resolved using non-reduced PAGE. Grass carp serum IgM migrated three protein bands larger than 170 kDa under non-reduced conditions (Figure 2B), which could represent variable polymerization into monomeric, dimeric, and tetrameric forms, as reported previously in common carp and Indian major carp (41, 42). A decrease in the molecular size after PNGase F digestion was apparent in the heavy chains of grass carp IgM, while the band corresponding to the light chain was not affected by PNGase F treatment (Figure 2C). This suggests that N-linked oligosaccharides are present on the grass carp IgM heavy chain but not on the light chain.

Bioinformatics analysis of the amino acid sequences of the light chain (GenBank No. AEH76780.1 and AEH76777.1) also indicated no N-linked carbohydrate attached to the grass carp IgM light chain (data not shown). Analysis indicated that each of the heavy chains contains four potential N-glycosylation sites restricted to the constant regions. Two of these are located at Asn-262 and Asn-303 in the CH2 domain, one at Asn-426 in the CH3 domain, and one at the Asn-565 residue within the conserved C-terminal tailpiece of the CH4 domain (Figure 2D).

### Site occupancy of four putative N-glycosylation sites

**Figure 3:**
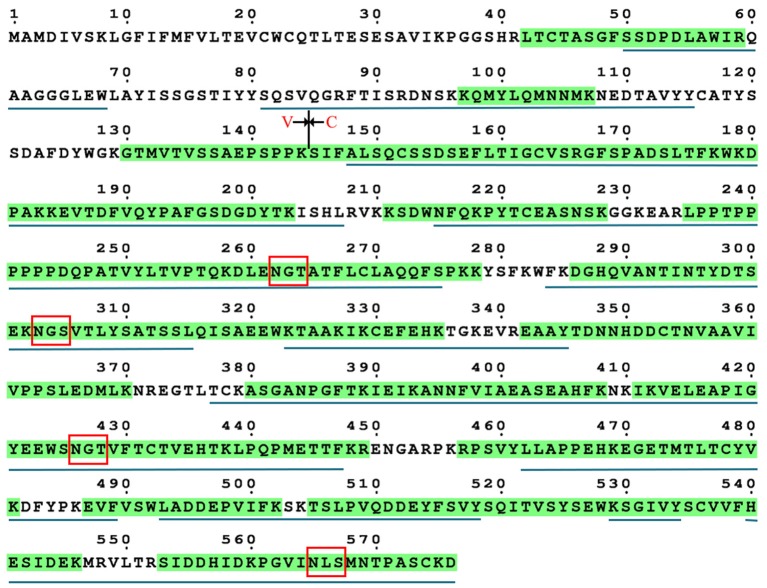
Sequence coverage of grass carp IgM heavy chain. The identified tryptic peptides are indicated by green shadow, four putative N-glycosylation sites are marked by red box. The identified chymotryptic peptides are indicated by black underline.

**Figure 4:**
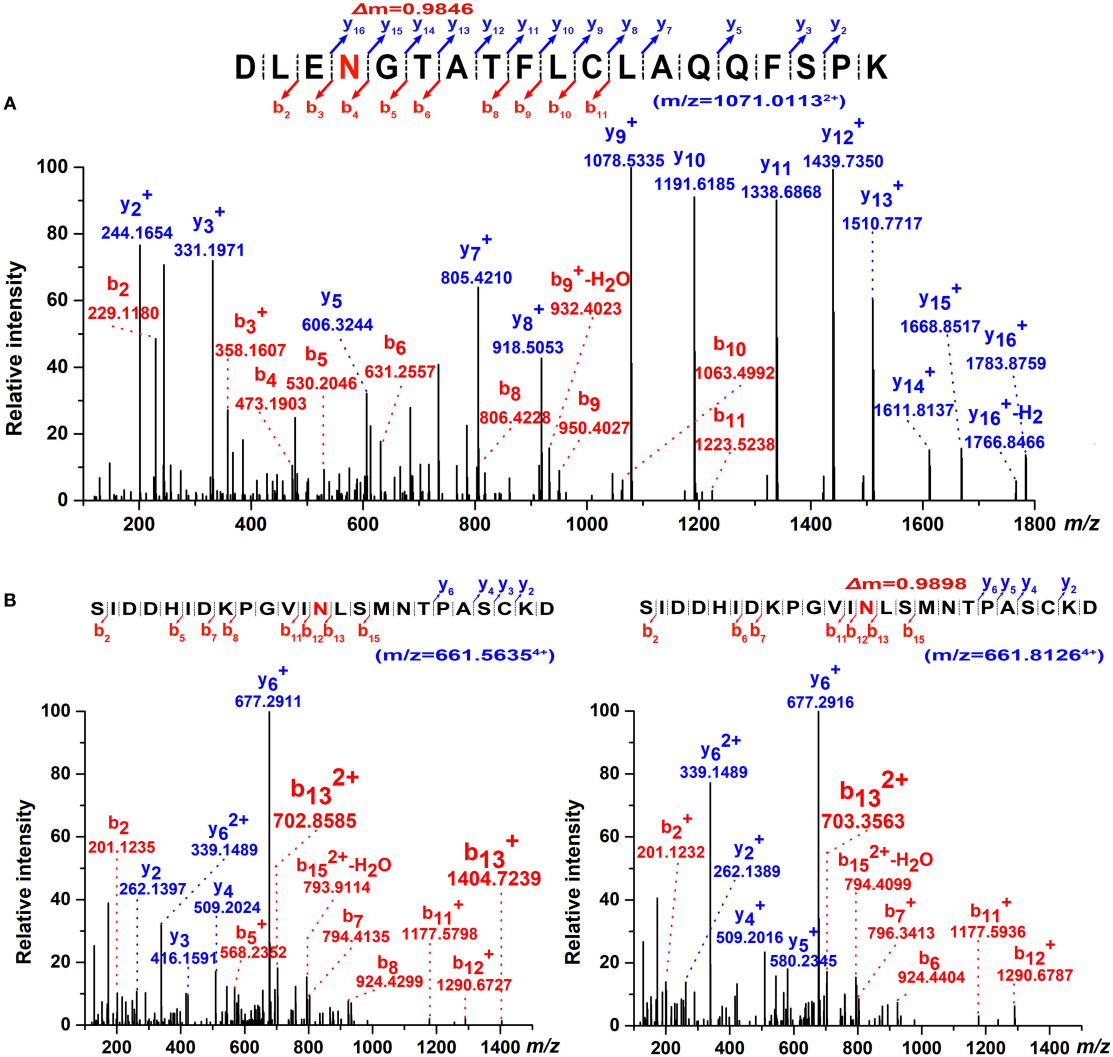
Determination of N-glycosylation sites by high-resolution ESI MS. **(A)** Representative MS/MS spectrum of the tryptic ^259^D–^277^Kpeptide (*m/z* = 1071.0113^2+^) covering Asn-262 glycosite with PNGase F digestion. The mass increment of 0.9846 Da comparing to its theoretical mass weight is indicated. **(B)** Dimer-isotopic mass spectrum of ^553^S–^576^D peptide covering Asn-565 glycosite. Non-glycosylated ^553^S–^576^D peptide (*m/z* = 661.5635^4+^) is shown in left panel, while de-glycosylated ^553^S–^576^D peptide (*m/z* = 661.8126^4+^) with a mass increasing of 0.9898 Da due to the converting of Asn-565 residue to an Asp with PNGase F treatment is indicated in right panel.

The occupancy at each glycosylation site was first checked via PNGase F digestion using LC-ESI-MS/MS. Treatment with PNGase F specifically releases N-glycans attached to the nitrogen of Asn, thereby converting Asn residue to Asp with mass increasing of 0.984 Da; thus, glycosylation sites can be deduced by amino acid sequence analysis after PNGase F treatment (30, 46, 47). Overall, tryptic peptides accounted for 72.74% of the total heavy chain amino acid sequence and 86.99% of the constant region, and the identified region spanning the four N-glycosylation sites on the heavy chain (Figure 3). The observed tryptic peptide masses derived from the Asn-262, Asn-303, and Asn-426 glycosylation sites exhibited increases of 0.9846–0.9850 Da compared with their theoretical mass weights, indicating that the Asn-262, Asn-303, and Asn-426 residues were glycosylated and converted to aspartic acid after PNGase F digestion (Figure 4A, Table 1). In contrast, both de-glycosylated and non-glycosylated Asn-565 sites were detected, with the tryptic peptide ^553^S–^575^K being observed in both forms in the dimer-isotopic mass (2512.2140 Da/2513.2232 Da) and with the additional oxidation at the methionine residue (2528.2104 Da/2529.2130 Da) (Table 1). Furthermore,^553^S–^576^D, with one trypsin digest missing, was also observed in the dimer-isotopic mass (2627.2428 Da/2628.2347 Da) and with methionine oxidation (2643.2383 Da/2644.2287 Da) (Figure 4B, Table 1), indicating that the Asn-565 residue is not completely utilized.

**Table 1:** ESI-MS/MS data observed fragments containing potential N-linked glycosylation sites.

**Peptide**	**Glycosite**	**Theoretical MW(Da)**	**Observed MW(Da)**	**Difference (Da)**
^259^D–^277^K	Asn-262	2140.0379^a^	2141.0225	0.9846
^303^N–^323^K	Asn-303	2271.1139	2272.0982	0.9843
^413^V–^438^K	Asn-426	2996.3982^a^	2997.3830	0.9848
^411^I–^438^K		3237.5773^a^	3238.5633	0.9850
^553^S–^575^K	Asn-565	2512.2170^a^	2512.2140	−0.0030
			2513.2232	1.0075
	Asn-566	2528.2120^a, b^	2528.2104	−0.0016
			2529.2130	1.0010
^553^S–^576^D	Asn-565	2627.2440^a^	2627.2428	−0.0012
			2628.2347	0.9907
	Asn-565	2643.2389^a^.^b^	2643.2383	−0.0006
			2644.2287	0.9898

a Peptide modifications with alkylation (Δm = 57.0215 Da) of the cysteine by iodoacetamide.

b *Peptide modifications with oxidation (Δm = 15.9994 Da) of the methionine*.

### Comparison of N-glycan occupancy at Asn-565 between monomeric, dimeric, and tetrameric IgM

**Figure 5:**
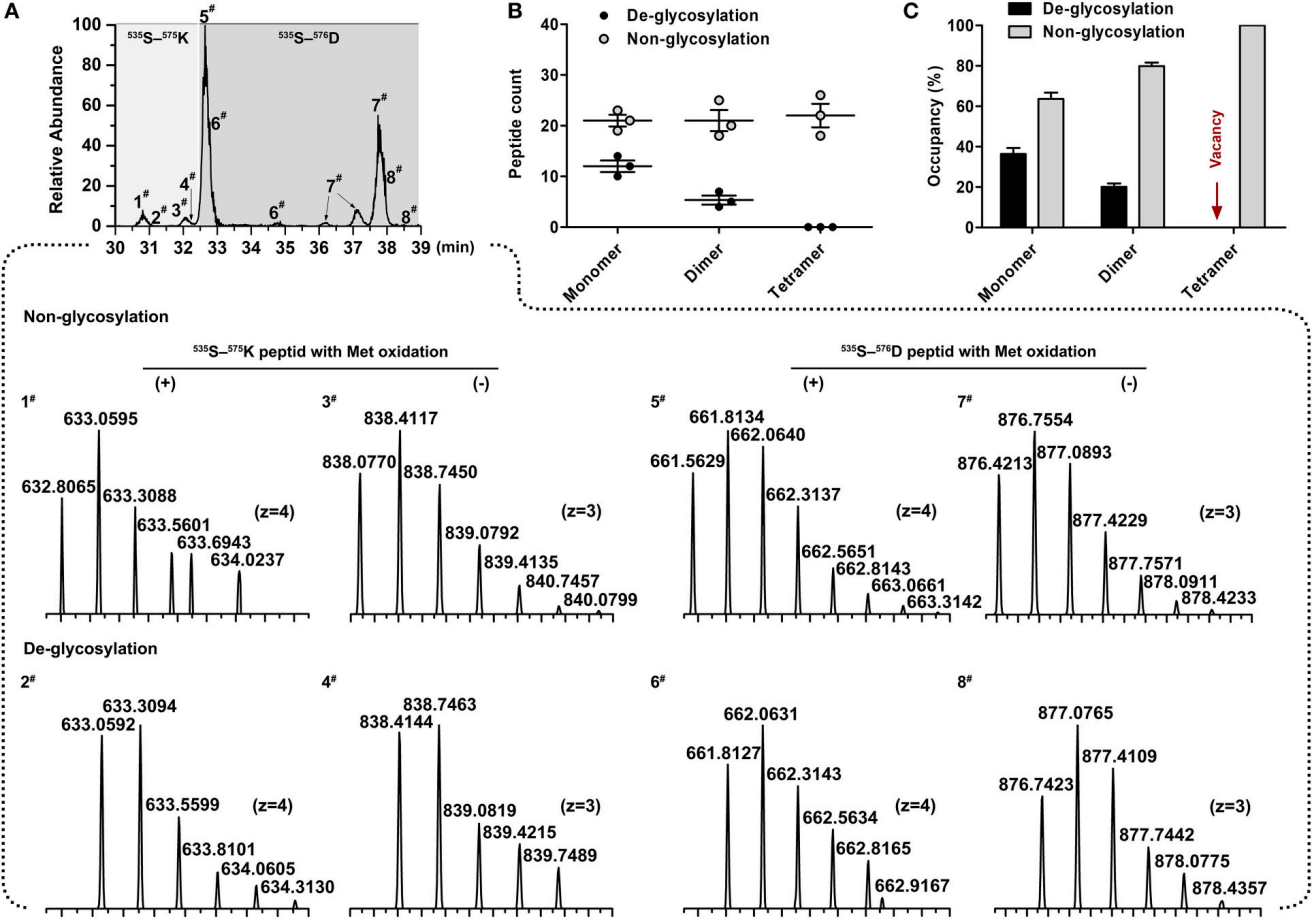
Determination of site occupancy for Asn-565 glycosite. **(A)** Overlaid chromatograms of non-glycosylated and de-glycosylated Asn-565 peptides. A total of 8 peptide variants were identified; 1^#^ and 3^#^, 5^#^ and 7^#^, non-glycosylated ^553^S–^575^K peptide and ^553^S–^576^D peptide without, and with methionineoxidation. 2^#^ and 4^#^, 6^#^ and 8^#^, De-glycosylated ^553^S–^575^K peptide and ^553^S–^576^D peptide without, and with methionineoxidation. **(B)** Asn-565 peptides spectral counts plot. Non-glycosylated or de-glycosylated Asn-565 peptides counts are shown by the sum of ^553^S–^575^K peptide and ^553^S–^576^D peptide. **(C)** Asn-565 glycosite occupancy. Occupancy was calculated by sum of the non-glycosylated or de-glycosylated peptide as a percentage of the total spectral counts of non-glycosylated and de-glycosylated peptide. Error bars in **(B,C)** indicate the standard deviation of the same serum pools with three repeats.

To assess the glycosylation occupancy at the Asn-565 site among different IgM isoforms, the monomeric, dimeric, and tetrameric IgM bands (seen in Figure 1B) were excised and de-glycosylated by PNGase F. Then, the occupancy of this site was estimated by comparing the relative amounts of de-glycopeptide and non-glycopeptide. Although isotopic clusters of PNGaseF de-glycosylated peptides (Δ*m* = 0.984 Da) could be clearly determined in comparison with non-glycosylated Asn-565 peptides via mass spectrometry (Figure 5A), some de-glycosylated peptide peaks overlapped with non-glycosylated peptides peak, making it difficult to estimate the peak areas between de-glycosylated and non-glycosylated Asn-565peptides. Occupation was then estimated by comparing the peptide spectral matches (PSMs) of de-glycosylated and non-glycosylated Asn-565 peptides. A striking decrease in de-glycosylated peptides was observed for dimeric IgM as compared with that for monomeric IgM, with the glycan occupancy at the Asn-565 site estimated to be approximately 36.34% for monomeric IgM and about 20.10% for dimeric IgM (Figures 5B,C). Moreover, no de-glycosylated Asn-565 peptide was detected on tetrameric IgM in three separate analyses, suggesting that Asn-565 is unoccupied by N-glycans on tetrameric IgM.

### Identification of glycopeptides

**Figure 6:**
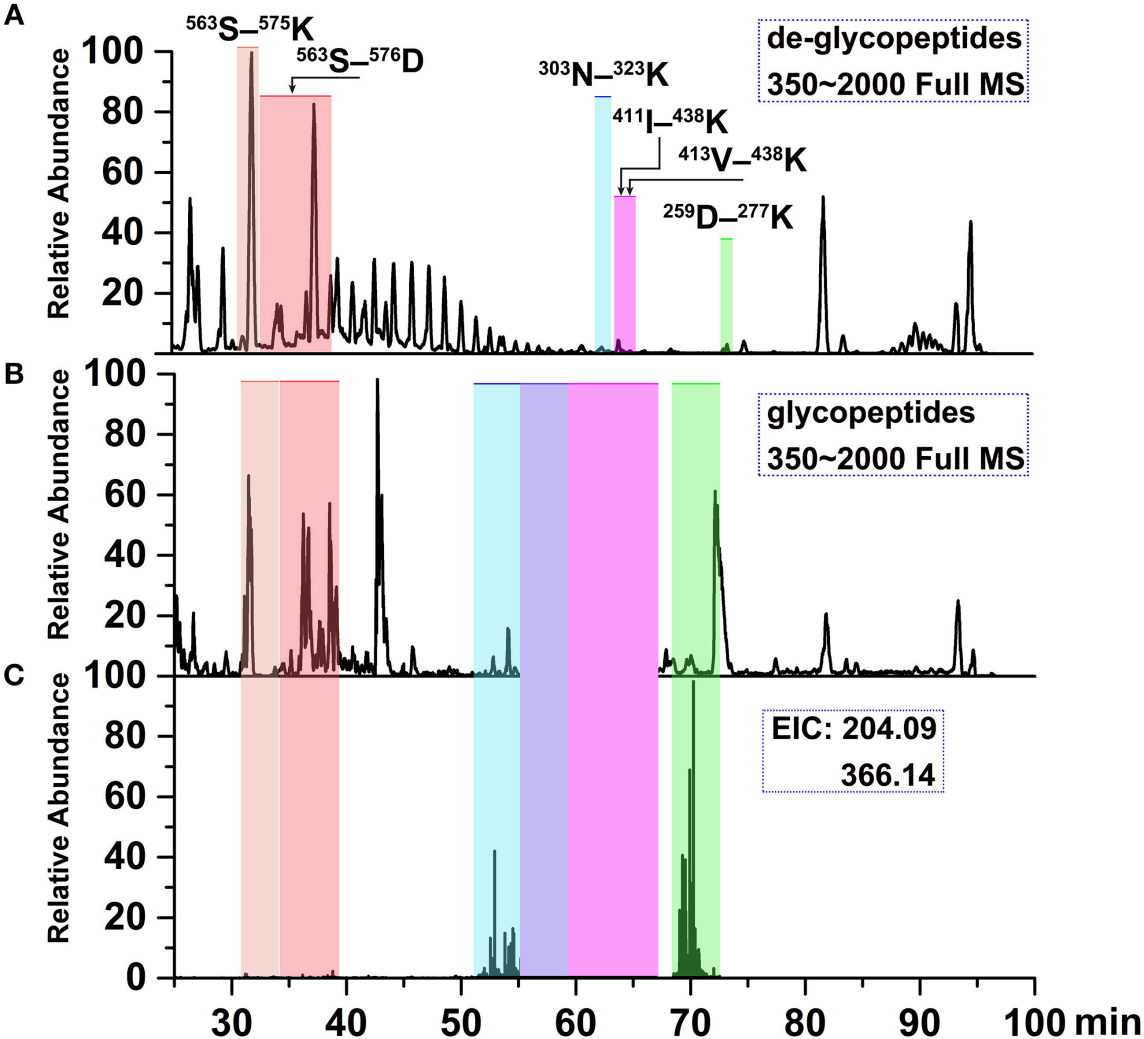
Ion chromatograms of LC-separated tryptic peptides. **(A)** Base peak chromatogram (BPC) of the tryptic de-glycopeptides with PNGase F digestion. **(B)** BPC of tryptic glycopeptides with HILIC enrichment. **(C)** Extracted ion chromatograms (EICs) of diagnostic oxonium fragment ions at *m/z* of 204.09 and 366.14. The shaded regions depict the identified deglycopeptides, glycopeptide clusters and Tables 2, 3 summarizes glycan heterogeneity at each site.

To determine site-specific glycan profiles at each of the four sites of the grass carp serum IgM, tryptic glycopeptides were investigated with LC-ESI-MS/MS by employing HCD fragmentation. Analysis of the grass carp IgM glycopeptides could provide site-specific glycans information. A typical LC-ESI-MS/MS base peak intensity chromatogram for individual tryptic glycopeptide family is shown in Figure 6. Compared with de-glycopeptide, individual glycopeptide family eluted over wider chromatographic intervals, which indicating that the retention time is influenced by the peptide and also modulated by the sugar moiety.

**Figure 7:**
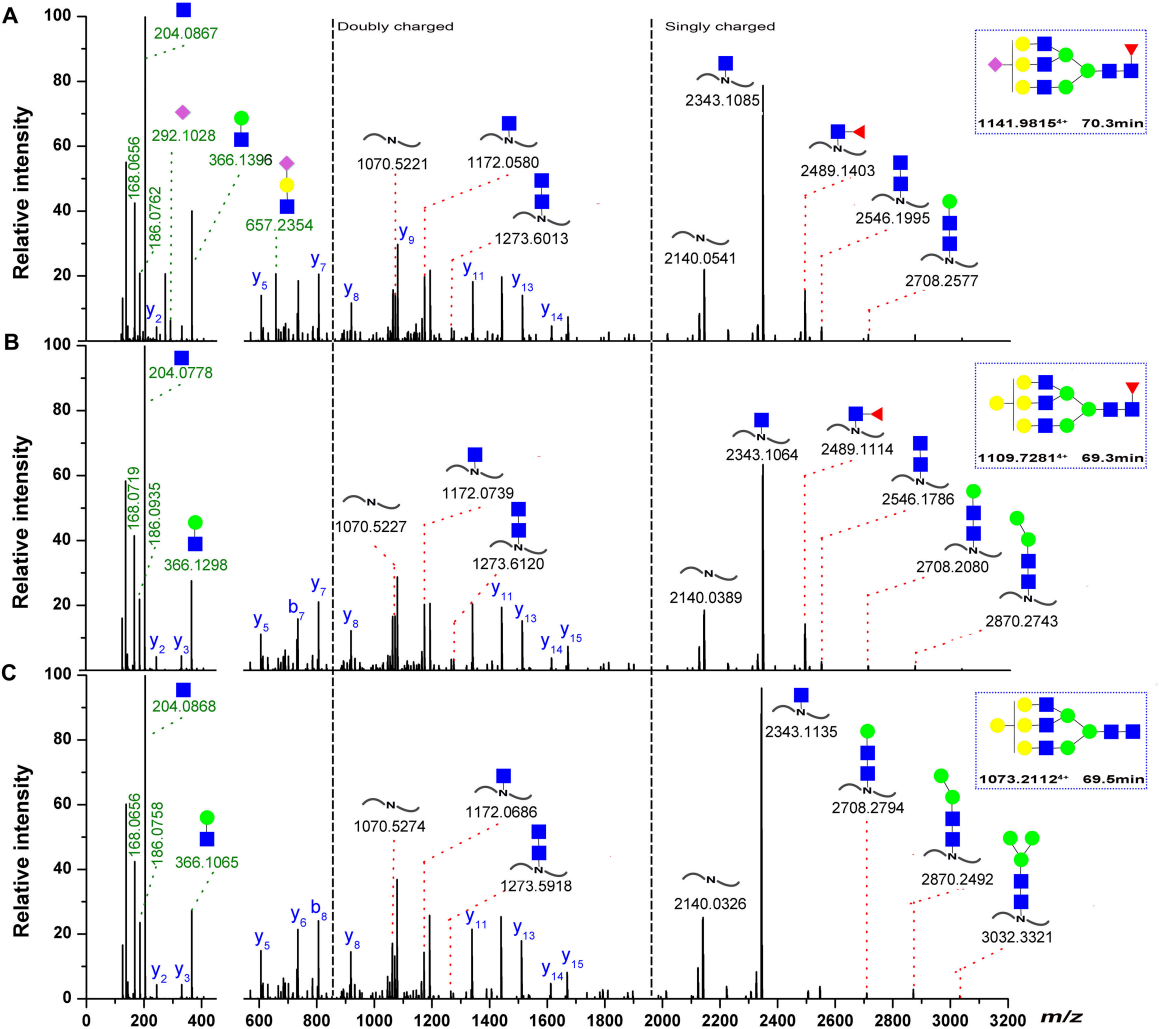
Representative spectra for Asn-262 glycopeptides with a sialylated glycan. **(A)** A neutral glycan with core-fucose **(B)** A neutral glycan without core-fucose glycan. **(C)** The oxonium ions were used to extract the intact glycopeptide spectrum, and the masses of the precursor and peptide/peptide + core GlcNAc ± Fuc ± Hex ions (red) can provide structural details, the b/y-ions (only y-ions were marked, blue) of the peptide portion were used to confirm peptide sequence. The proposed glycan structures were determined on the basis of monosaccharide composition, the monosaccharide linkages were not yet determined. Symbols as the following: blue box, N-acetylglucosamine; green circle, mannose; yellow circle, galactose; red triangle, fucose; pink diamond, N-acetylneuraminic acid.

Three representative glycans detected on the ^259^D–^277^K (Asn-262) peptide are shown in Figure 7. The oxonium ionsat *m/z* 204.08 (HexNAc^+^), 186.07 (HexNAc-H_2_O^+^), 168.06 (HexNAc-2H_2_O^+^), and 366.14 (HexNAc-Hex^+^) are readily discernible within the fragmentation pattern. The composition of each glycan was identified by data-dependent MS/MS with an accuracy of 10 ppm or less. On the basis of previous information regarding fish IgM N-glycosylation (25, 48), the deoxyhexose residue was recognized as fucose in this study. Sialylated ions, such as those at *m/z* 292.09 (NeuAc^+^) and *m/z* 657.23 (NeuAc-Hex-HexNAc^+^), indicated the presence of sialylated glycans, with fragment ions corresponding to core-fucose on the peptide+HexNAc residue suggesting core-fucosylated glycans (Figures 7A,B). Glycan sequencing results for HexNAc-Hex-NeuAc, peptide+HexNAc, peptide+2HexNAc, peptide+HexNAc+Hex, as well as the intact peptide backbone and a set of b/y ions, confirmed the assignment of the glycan to the ^259^D–^277^K tryptic peptide fragment (Figures 7A–C). Notably, the fucose on the core-HexNAc residue, observed as a peptide+HexNAc+Fucion, was unique fucose residue observed by MS/MS, indicating the location of the fucose as a core-fucosylation on N-glycans. Glycopeptide identification also revealed that *N*-acetylneuraminic acid (NeuAc), rather than *N*-glycolylneuraminic acid (NeuGc), was the only form of sialic acid observed in grass carp serum IgM.

### Site-specific N-glycan analysis

Assignments a set of glycans at each glycosite were achieved by selectively detecting tryptic glycopeptides, as shown in Tables 2, 3. Substantial heterogeneity was evident, with different glycan compositions detected at the four glycosites of grass carp serum IgM, including 22 forms at Asn-262, 15 at Asn-303, and 17 at Asn-426, indicating a considerable overlap in the glycoforms present at these three sites. In contrast, the Asn-565 glycosite was unique in having trace carbohydrate moieties and only three classes of glycans. In addition to trypsin, chymotrypsin digestion was also performed to increase sequence coverage and obtain more glycan information at each glycosite. The majority of glycans identified by chymotrypsin treatment were the same as those identified by trypsin digestion at the Asn-262, Asn-303, and Asn-426 glycosites, serving as a secondary verification of the site-specific glycosylation mapping (Table 4). Furthermore, chymotryptic glycopeptide analysis revealed 2–5 new glycans at each of the four glycosites, as shown in Table 4.

**Table 2:** Summary of N-glycans identified at Asn-262 and Asn-303 glycosites.

**Site**	**Theoretical glycopeptide (Da)**	**Observed glycopeptide (Da)**	**Mass error (ppm)**	**Glycans assignment**	**Chymo-trypsin support**	**Glycan type**
Asn-262	^259^D–^277^K Peptide only 2140.0379					
	5545.2383	5545.2804	7.6	HexNAc_6_Hex_9_Fuc_1_NeuAc_2_		Complex
	5416.1957	5416.2223	4.9	HexNAc_6_Hex_10_Fuc_1_NeuAc_1_		Complex
	5287.1531	5287.1681	2.8	HexNAc_6_Hex_11_Fuc_1_	√	Complex
	4946.0321	4946.0504	3.7	HexNAc_6_Hex_8_NeuAc_1_	√	Complex
	4816.9895	4816.9915	0.4	HexNAc_6_Hex_9_	√	Complex
	4783.9793	4783.9562	−4.8	HexNAc_6_Hex_7_NeuAc_1_		Complex
	4762.0215	4762.9993	−4.6	HexNAc_9_Hex_4_Fuc_1_		Complex
	4759.9681	4759.9622	−1.2	HexNAc_5_Hex_9_Fuc_1_		Complex
	4726.9578	4726.9458	−2.5	HexNAc_5_Hex_7_Fuc_1_NeuAc_1_	√	Complex
	4615.9635	4615.9572	−1.4	HexNAc_9_Hex_4_		Complex
	4597.9153	4597.9271	2.6	HexNAc_5_Hex_8_Fuc_1_		Complex
	4564.9050	4564.9038	−1.0	HexNAc_5_Hex_6_Fuc_1_NeuAc_1_	√	Complex
	4492.8839	4492.8909	1.6	HexNAc_6_Hex_7_	√	Complex
	4453.9107	4453.8905	−4.5	HexNAc_9_Hex_3_		Complex
	4435.8624	4435.8781	3.5	HexNAc_5_Hex_7_Fuc_1_	√	Complex
	4418.8471	4418.8540	1.6	HexNAc_5_Hex_6_NeuAc_1_	√	Complex
	4289.8045	4289.8220	4.1	HexNAc_5_Hex_7_	√	Complex
	4215.7677	4215.8026	8.3	HexNAc_4_Hex_6_NeuAc_1_		Complex
	4127.7517	4127.7724	5.0	HexNAc_5_Hex_6_	√	Complex
	3908.6774	3908.6958	4.7	HexNAc_4_Hex_5_Fuc_1_		Complex
	3762.6195	3762.6502	8.2	HexNAc_4_Hex_5_	√	Complex
	3600.5667	3600.5982	8.7	HexNAc_4_Hex_4_		Complex
Asn-303	^303^N–^323^K Peptide only 2271.1139					
	5078.0939	5078.0901	−0.7	HexNAc_6_Hex_8_NeuAc_1_	√	Complex
	4948.0656	4948.0675	0.4	HexNAc_6_Hex_9_	√	Complex
	4786.5831	4786.5823	−0.6	HexNAc_5_Hex_9_		Complex
	4729.9750	4729.9852	2.2	HexNAc_5_Hex_8_Fuc_1_	√	Complex
	4695.9810	4695.9827	0.4	HexNAc_5_Hex_6_Fuc_1_NeuAc_1_	√	Complex
	4624.9439	4624.9753	6.8	HexNAc_6_Hex_7_		Complex
	4584.9867	4584.9779	−1.9	HexNAc_9_Hex_3_		Complex
	4582.9334	4582.9300	−0.7	HexNAc_5_Hex_8_		Complex
	4566.9384	4566.9368	1.5	HexNAc_5_Hex_7_Fuc_1_	√	Complex
	4549.9231	4549.9228	−0.1	HexNAc_5_Hex_6_NeuAc_1_	√	Complex
	4420.8805	4420.8802	−0.1	HexNAc_5_Hex_7_	√	Complex
	4259.8120	4259.8116	−0.2	HexNAc_5_Hex_6_	√	Complex
	4097.7590	4097.7455	−3.3	HexNAc_5_Hex_5_	√	Complex
	3893.6955	3893.7251	7.6	HexNAc_4_Hex_5_	√	Complex
	3732.6270	3732.6080	−5.1	HexNAc_4_Hex_4_	√	Complex

*HexNAc, N-acetylglucosamine; Hex, hexose (mannose and galactose); Fuc, fucose; NeuAc, neuraminic (sialic) acid*.

**Table 3:** Summary of N-glycans identified at Asn-426 and Asn-565 glycosite.

**Site**	**Theoretical glycopeptide (Da)**	**Observed glycopeptide (Da)**	**Mass error (ppm)**	**Glycans assignment**	**Chymo-trypsin support**	**Glycan type**
Asn-426	^411^I–^438^K Peptide only 3237.5773					
	6384.6924	6384.7130	3.2	HexNAc_6_Hex_11_Fuc_1_		Complex
	6244.6352	6243.6729	6.6	HexNAc_5_Hex_6_Fuc_1_NeuAc_3_		Complex
	6043.5714	6043.6159	7.4	HexNAc_6_Hex_8_NeuAc_1_		Complex
	5914.5288	5914.5830	9.2	HexNAc_6_Hex_9_	√	Complex
	5662.4443	5662.4577	2.4	HexNAc_5_Hex_6_Fuc_1_NeuAc_1_		Complex
	5588.4075	5588.4274	3.6	HexNAc_4_Hex_5_Fuc_1_NeuAc_2_		Complex
	5549.3966	5549.4289	5.8	HexNAc_5_Hex_8_	√	Complex
	5533.4017	5533.4010	−0.1	HexNAc_5_Hex_7_Fuc_1_	√	Complex
	5516.3864	5516.3737	−2.3	HexNAc_5_Hex_6_NeuAc_1_	√	Complex
	5475.3599	5475.3593	−0.1	HexNAc_4_Hex_7_ NeuAc_1_		Complex
	5387.3438	5387.3392	−0.9	HexNAc_5_Hex_7_	√	Complex
	5313.3070	5313.3283	4.0	HexNAc_4_Hex_6_NeuAc_1_	√	Complex
	5225.2910	5225.3392	9.2	HexNAc_5_Hex_6_	√	Complex
	5192.2808	5192.3053	4.7	HexNAc_5_Hex_4_NeuAc_1_	√	Complex
	5184.2645	5184.2809	3.2	HexNAc_4_Hex_7_	√	Complex
	5063.2382	5063.2414	0.6	HexNAc_5_Hex_5_	√	Complex
	4965.1902	4965.2057	3.1	HexNAc_3_Hex_6_Fuc_1_		Hybrid/Complex
	^413^V–^438^K Peptide only 2996.3982					
	5802.3924	5802.3942	0.3	HexNAc_6_Hex_8_NeuAc_1_		Complex
	5308.2176	5308.2433	4.8	HexNAc_5_Hex_8_	√	Complex
	5292.2227	5292.2443	4.1	HexNAc_5_Hex_7_Fuc_1_	√	Complex
	5275.2074	5275.2091	0.3	HexNAc_5_Hex_6_NeuAc_1_	√	Complex
	5146.1648	5146.1955	6.0	HexNAc_5_Hex_7_	√	Complex
	5072.1280	5072.1532	5.0	HexNAc_4_Hex_6_NeuAc_1_		Complex
	4822.0592	4822.0867	5.7	HexNAc_5_Hex_5_	√	Complex
Asn-565	^553^S–^575^K Peptide only 2512.2170					
	4587.9468	4587.9451	−0.4	HexNAc_4_Hex_6_NeuAc_1_		Complex
	^553^S–^576^D Peptide only 2627.2440					
	5052.1111	5052.1203	1.8	HexNAc_5_Hex_6_Fuc_1_NeuAc_1_		Complex
	4777.0106	4777.0028	−1.6	HexNAc_5_Hex_7_	√	Complex

*For abbreviations, see Table 2*.

**Table 4:** Summary of N-glycans mapping by chymotrypsin treatment.

**Sites**	**Glycans assignment^a^**	**Glycan type**
Asn-262	HexNAc_6_Hex_8_Fuc_1_NeuAc_1_	Complex
	HexNAc_5_Hex_8_	Complex
	HexNAc_5_Hex_6_Fuc1	Complex
	HexNAc_5_Hex_5_	Complex
Asn-303	HexNAc_5_Hex_6_NeuAc_2_	Complex
	HexNAc_5_Hex_5_NeuAc_2_	Complex
	HexNAc_5_Hex_6_Fuc_1_	Complex
	HexNAc_5_Hex_5_NeuAc_1_	Complex
	HexNAc_4_Hex_4_Fuc_1_	Complex
Asn-426	HexNAc_6_Hex_7_	Complex
	HexNAc_4_Hex_6_	Complex
	HexNAc_5_Hex_4_	Complex
Asn-565	HexNAc_5_Hex_6_NeuAc_1_	Complex
	HexNAc_5_Hex_6_	Complex

a *New detected glycoform in each glycosite are indicated by gray shadow. Abbreviations used, see Table 2*.

Of the 38 total glycan compositions observed on grass carp serum IgM by tryptic and chymotryptic glycopeptide analysis, 17 were sialylated and 21 were not. Notably, when the glycans were grouped into high-mannose, hybrid, complex types, the glycans identified on grass carp serum IgM were limited to complex-type carbohydrates, with the exception of a single carbohydrate with a HexNAc_3_Hex_6_Fuc_1_ composition on Asn-426 glycosite, for which the identification as a hybrid or complex type glycan was ambiguous. Heterogeneity was greatest at the Asn-262 glycosite, with both neutral and mono/disialylated, as well as small and large glycans, were observed. The Asn-303 and Asn-426 glycosites exhibited the next highest levels of variation. Three novel oligosaccharides, HexNAc_9_Hex_3_, HexNAc_9_Hex_4_, and HexNAc_9_Hex_4_Fuc_1_, with redundancy in the HexNAc monosaccharide (tentatively assigned as GalNAcβ1 → 4GlcNAc antennae, the relatively rare lacdiNAc motif) were identified on grass carp serum IgM with a mass accuracy within 5 ppm. No other glycan composition was possible with respect to the observed *m/z* values, suggesting the presence of glycans on grass carp serum IgM containing the diacetyllactosamine motif. Unlike IgMs in other higher vertebrates and some reported fish species, which possess major high-mannose glycans at the C-terminal tailpiece glycosite, the Asn-565 site of the grass carp serum IgM possesses exclusively complex-type N-glycans and is devoid of high-mannose structures. Together, these data indicate that the glycans on grass carp serum IgM are highly processed. It should also be pointed out that only a minority of Asn-565 peptides were conjugated with carbohydrates with both tryptic and chymotryptic glycopeptide analysis, further confirming the incomplete glycan occupancy at the Asn-565 glycosite.

### MALDI-TOF MS analysis of N-glycans

**Figure 8:**
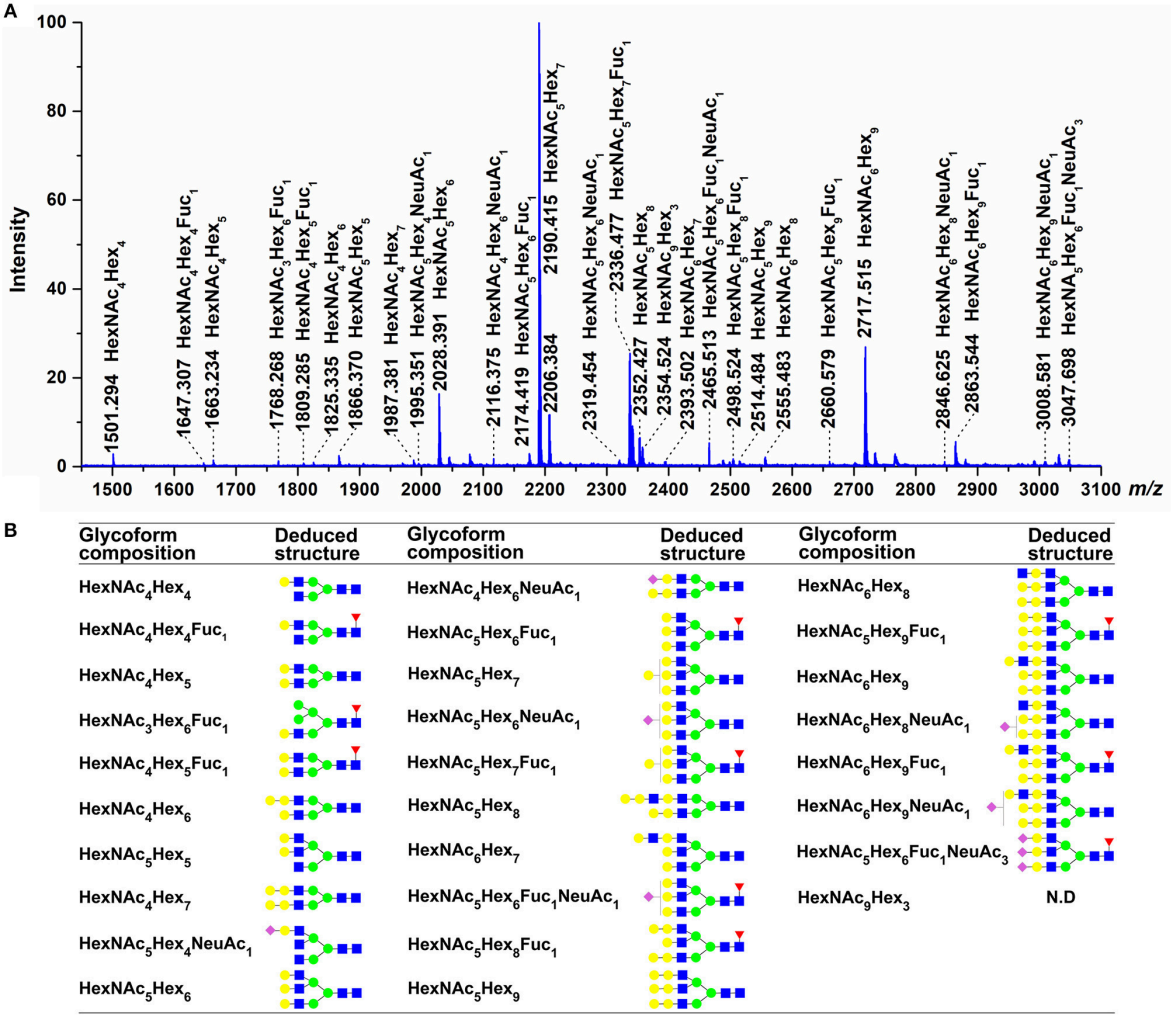
Glycans detected by MALDI-TOF. **(A)** Molecular masses are of unlabeled glycans, detected as [M + Na]^+^ in positive polarity mode. **(B)** The proposed glycan structures were determined on the basis of monosaccharide composition, as well as literature (24, 25, 48). For symbol definitions, seen Figure 6. N.D., not determined.

Information from released glycans can be used to confirm the results of glycopeptide analysis and provide additional insight into the N-glycans present on grass carp serum IgM. The MALDI-TOF spectra of the released glycans revealed that the main carbohydrate moieties are HexNAc_3−6_Hex_4−9_Fuc_1_NeuAc_1,3_ complex-type glycans (Figure 8), which is in general agreement with the IgM glycopeptide analysis. Of these, the four most abundant peaks corresponded to HexNAc_5_Hex_7_, HexNAc_6_Hex_9_, HexNAc_5_Hex_7_Fuc_1_, and HexNAc_5_Hex_6_ glycan compositions. These four glycans were also prominent among those observed in the glycopeptide analysis, as HexNAc_5_Hx_7_ presented at each of the four glycosites; HexNAc_6_Hex_9_ and HexNAc_5_Hex_7_Fuc_1_ appeared at the Asn-262, Asn-303, and Asn-426 sites; and HexNAc_5_Hx_6_ appeared at the Asn-262 and Asn-426 sites. Some sialylated glycan structures, such as HexNAc_4−5_Hex_4−6_Fuc_0−1_NeuAc_1,3_, were detected by MALDI-TOF analysis. Notably, the HexNAc_4_Hex_6_, HexNAc_6_Hex_8_, and HexNAc_6_Hex_9_Fuc_1_ glycans, which were observed by MALDI-TOF but not glycopeptide analysis, may be, to some extent, artifactually generated forms of the HexNAc_4_Hex_6_NeuAc_1_, HexNAc_6_Hex_8_NeuAc_1_, and HexNAc_6_Hex_9_ Fuc_1_NeuAc_1_ glycans, respectively, derived from the loss of sialic acid residues during the MALDI analysis. Data from the released glycans also provided additional information not present in the glycopeptide analysis, as a HexNAc_6_Hex_9_Fuc_1_ glycan with an *m/z* >2800 was detected by MALDI-TOF MS. Overall, MALDI-TOF analysis of released glycans further confirmed that the main oligosaccharides present on grass carp serum IgM are complex-type glycans.

## Discussion

In this study, a detailed site-specific N-glycan profile and glycan occupancy at the C-terminal tailpiece glycosite were described for the first time of the teleost IgM. The results revealed that grass carp serum IgM possesses primarily complex-type oligosaccharides that may be either neutral or sialylated. Glycan occupancy at the C-terminal tailpiece glycosite has significant different among grass carp IgM isoforms.

The N-linked carbohydrate moieties of carp IgM were found to be restricted to the heavy chain. The Asn-262 and Asn-303 glycosites in the CH2 domain and Asn-426 glycosite in CH3 were found to be fully occupied, while the Asn-565 site in the C-terminal tailpiece was incompletely occupied. It is a structural feature of teleost IgMs that a number of teleosts produce heterogeneous mixtures of IgM polymers. This has been attributed to the variation in the degree of disulfide polymerization, referred to as redox forms, and has implications for teleost IgM assembly processes (26, 49). Here, we found that the occupancy at the Asn-563 site is markedly different between monomeric and dimeric IgM forms, while the site is totally unoccupied in tetrameric IgM. This is the first evidence of variable site occupancy at the C-terminal tailpiece of teleost IgM isoforms.

In the site-specific analysis, the largest heterogeneity in oligosaccharides was observed at the Asn-262 glycosite, followed by Asn-303 in the CH1 domain and Asn-426 in the CH3 domain. The main oligosaccharides were found to be complex-type glycans with considerable heterogeneity, with neutral; monosialyl-, disialyl-, and trisialylated; and fucosyl-and non-fucosyl-oligosaccharides conjugated to these three sites. Interestingly, oligosaccharides with HexNAc_9_Hex_3_, HexNAc_9_Hex_4_Fuc_1_, and HexNAc_9_Hex_4_ compositions with redundancy in the HexNAc monosaccharide were identified at the Asn-262 and Asn-303 glycosites by glycopeptide analysis. This indicates that Asn-262 and Asn-303 are occupied by glycans with terminal HexNAc residues, possibly lacdiNAc motifs (GalNAcβ1 → 4GlcNAcantennae), as are common on invertebrate glycans as well as the N-glycans of zebrafish embryos (48, 50, 51). Compared to the C-terminal tailpieces of glycosites in higher vertebrates, which possess major high-mannose glycans (30, 43, 52), the C-terminal tailpiece glycosite of grass carp serum IgM possesses exclusively complex-type N-glycans and is devoid of high-mannose glycans. Although the minority of Asn-565 glycosites were conjugated with carbohydrates, grass carp serum IgM possesses sialylated oligosaccharides at the C-terminal tailpiece glycosite.

Previous studies of the oligosaccharides on the C-terminal tailpiece have documented their important roles in determining IgM polymerization in mammals (38). It has been found that human IgM polymers are influenced by the presence of glycans linked to Asn-563, as replacement of Asn-563 with Ala abolishes the attachment of glycan moieties and results in the secretion of hexameric and higher molecular weight species (39). It has been suggested that glycans linked to Asn-563, located in the tightly packed IgM core, may limit the number of subunits that can be incorporated into an oligomer or serve as a docking device for ERGIC53, a hexameric lectin shown to promote polymerization in non-lymphoid cells (53). Teleost IgM lacks J chain and tetrameric IgM was thought to polymerize by interchain disulfide linkages (26). Our observation of highly processed oligosaccharides and variable site occupancies at the C-terminal tailpiece glycosite of grass carp IgM isoforms may therefore have important biological implications. It may indicate that glycans linked to the C-terminal tailpiece have an impact on teleost IgM oligomer formation, and oligosaccharides presenting on the C-terminal tailpiece can interfere teleost IgM oligomer formation.

Glycosylation is highly susceptible to changes of the physiological conditions and environmental factors (54), changes related to age, gender, and pregnancy have also been described for mammalian Ig glycan (55, 56). Various B-cell stimuli have been shown to modulate Ig glycosylation, as CpG oligodeoxynucleotide and IL- 21 have been proved to increase Fc galactosylation and reduce bisecting GlcNAc levels. While, all-trans retinoic acid decreases galactosylation and sialylation levels (57). The transcription factor hepatocyte nuclear factor (HNF) 1a and its downstream target HNF4a have been identified as critical regulators of fucosyltransferase and fucose biosynthesis genes, thus influence N-glycan levels in plasma protein (58). It is known teleost produce both mucosal (mlg) and serum (slg) IgM. Previous report indicated glycosylation patterns may differences between mlgM and slg IgM, as mAb reacting with mlgM H chain carbohydrate determinant not or poorly with slg, which indicating differences in carbohydrate composition or structure (41). Knowledge on teleost mlgM glycosylation is scant so far, analysis of mIgM are expected to shed light on the mlg mucosa-associate Ig glycosylation pattern.

It has been found that differiental of sugar moiety influences mammalian antibody affinity to activating or inhibitory FcγR and thus influence antibody effector activity. IgG galactosylation has been found to be decreased in rhumatoid arthritis, and galactosylated IgG correlates with remission of arthritis during pregnancy rheumatoid arthritis patients (56, 59, 60). Changes in mouse IgG glycosylation are considered to affect the binding affinity of IgG to FcγR (61, 62). Absence of core fucose in human IgG was reported to alter binding affinity to FcγRIII, the effect of therapeutic anticancer mAb as trastuzumab with decreased fucosylation has been well recognized and is currently being harnessed in clinical trials (63, 64). Attachment of sialic acid on IgG appears to mediate the anti-inflammatory effects, in contrast, a lack of terminal sialic acid residue appears to mediate the inflammatory effect leading to osteoclast differentiation and bone loss by increases the affinity to activating FcγRIIand FcγRIII (65, 66). The N-glycans on IgM are of particular interest because IgM has a relatively high carbohydrate content compared with those of other immunoglobulin classes. Until now, very little has been known specifically about the role of the carbohydrate moiety in fish IgM. Thus, it would be of considerable interest to determine whether teleost IgM N-linked glycans have functional roles in interactions with natural biological partners, such as complement system components and/or FcR on immune cells. Work along these lines is now in progress in our laboratory.

In conclusion, our study highlights the importance of performing site-specific characterization of glycoproteins at multiple glycosites, as total glycosylation characterization of the entire protein will fail to reveal differences in the glycosylation distributions of individual sites. The site-specific characterization of the N-glycans of teleost IgM described here should facilitate targeted partial de-glycosylation approaches for subsequent functional analysis in order to obtain further insight into the functional effects of glycosylation on teleost IgM.

## Data availability statement

The RAW MS data of grass carp serum IgM analysis, as well as the original search results of pGlyco 2.0 have been uploaded to the PRIDE partner repository (https://www.ebi.ac.uk/pride/archive/) with access codes: PXD010308.

## Author contributions

HG, Y-AZ, and BW conceived and designed the experiments. Y-LS, M-DH, Z-WC, and JW performed the experiments. Y-LS, QY, and HB analyzed the data. HG, Y-FC, LX, and C-HW wrote the manuscript. All authors reviewed the manuscript.

### Conflict of interest statement

The authors declare that the research was conducted in the absence of any commercial or financial relationships that could be construed as a potential conflict of interest.
